# Image fusion–guided percutaneous transthoracic embolization of ascending aortic pseudoaneurysm

**DOI:** 10.1016/j.xjtc.2022.03.006

**Published:** 2022-04-12

**Authors:** Antara Dattagupta, Pauline M. Berens, Mahesh K. Ramchandani, Alan B. Lumsden, Ponraj Chinnadurai, Moritz C. Wyler von Ballmoos

**Affiliations:** a Texas A&M College of Medicine, Bryan, Tex; b DeBakey Heart & Vascular Center, Houston Methodist Hospital, Houston, Tex; c Stanford University Medical Center, Stanford, Calif; d Advanced Therapies, Siemens Medical Solutions USA, Inc, Malvern, Pa

**Figure:**
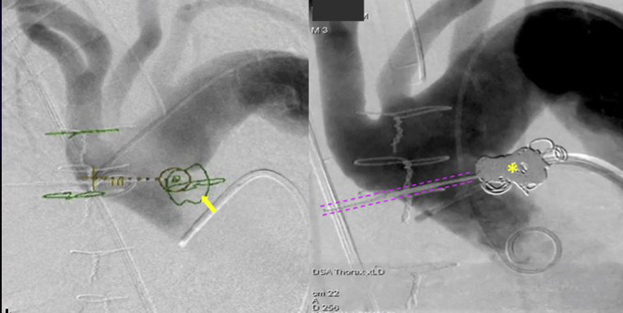
Percutaneous, transthoracic coil-embolization of an ascending aortic pseudoaneurysm.

**Central Message**

We demonstrate the use of percutaneous, transthoracic coil-embolization of an aortic pseudoaneurysm with the help of advanced image fusion as an alternative to described endovascular solutions (Video 1).

A 77-year-old frail woman with a 5.1-cm ascending aortic aneurysm, enlarged aortic root, and severe aortic regurgitation (Figure 1, *A*) underwent an uneventful valve-sparing aortic root replacement (David V procedure) and ascending aorta replacement. Postoperative transthoracic echocardiogram demonstrated a competent aortic valve with a coaptation zone of 8 mm. She was discharged on postoperative day 7. One month later, the patient presented after a syncopal episode. Further workup including cardiac computed tomography angiography (CCTA) revealed an area of contrast extravasation at the proximal lesser curvature of the aortic arch. The diagnosis of a distal anastomotic leak with formation of a pseudoaneurysm compressing the left main pulmonary artery was made (Figure 1, *B*).

**Figure 1:**
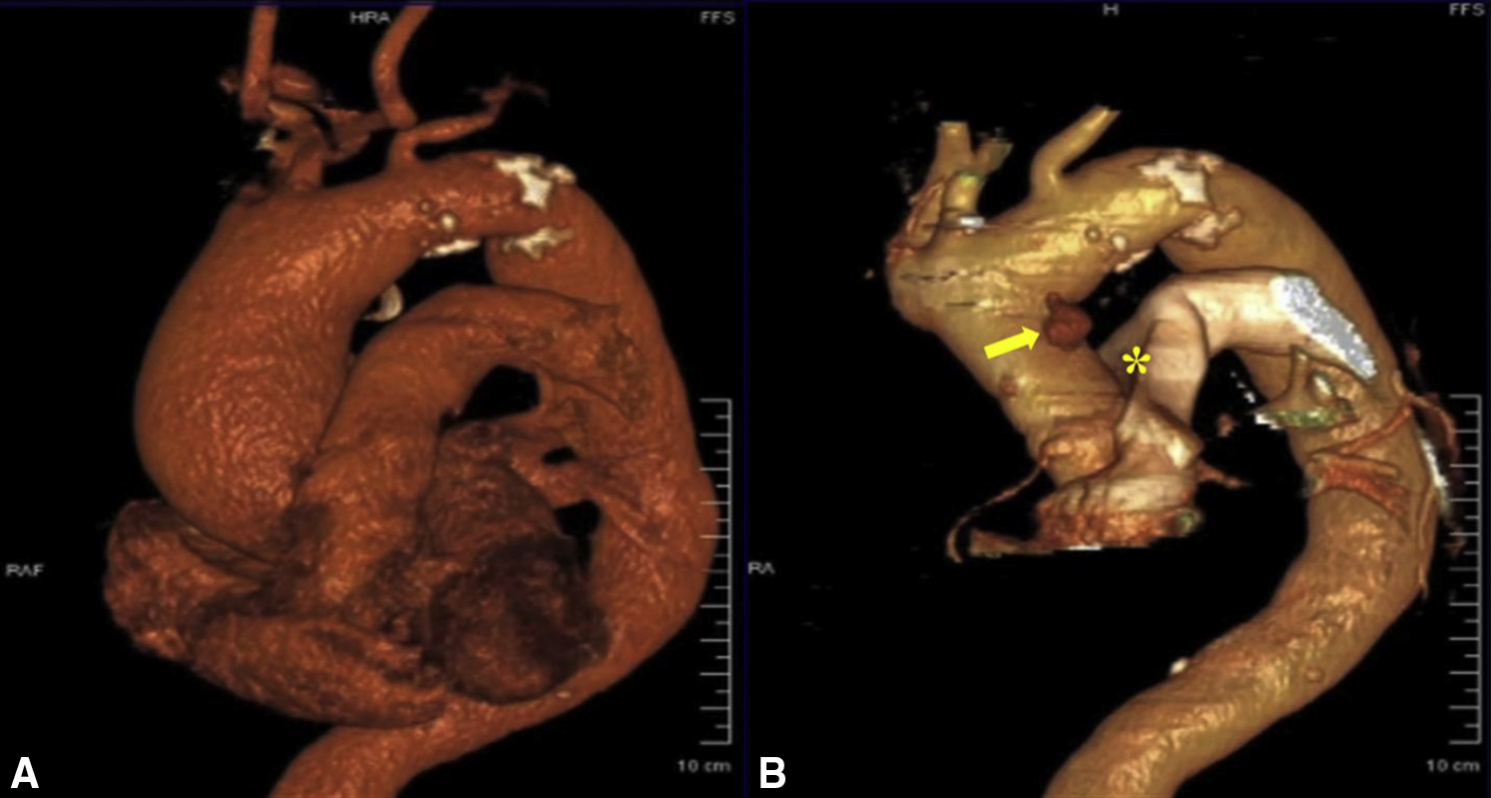
Ascending aortic aneurysm repair. A, On admission, preoperative cardiac computed tomography angiography demonstrated a 5.1-cm thoracic ascending aortic aneurysm. B, One month postoperatively, imaging showed a distal anastomotic leak with pseudoaneurysm formation (*arrow*) compressing the left main pulmonary artery (*asterisk*).

In light of recent surgery and significant frailty, a less-invasive, nonsurgical treatment option was felt to be desirable for this patient. Endovascular solutions for the treatment of ascending aortic pseudoaneurysms have previously been described.^1^, ^2^, ^3^, ^4^ A covered stent was considered but not feasible, given the proximity of the pseudoaneurysm to the head vessels with an insufficient landing zone. Endovascular embolization of the ascending aortic pseudoaneurysm (AAP) was attempted using both transfemoral and transbrachial access but was unsuccessful due to the small neck of the AAP arising from the distal suture line and poor opacification of the AAP on angiography. Repeat CCTA following a failed endovascular attempt showed an enlarging AAP with increased contrast extravasation in the peri-aortic region (Figure 2, *A* and *B*). The patient was then taken to a hybrid operating room equipped with a robotic-assisted angiography system (ARTIS pheno VE10; Siemens Healthineers) for direct, percutaneous, image fusion–guided embolization of her AAP with cardiopulmonary bypass on standby.

**Figure 2:**
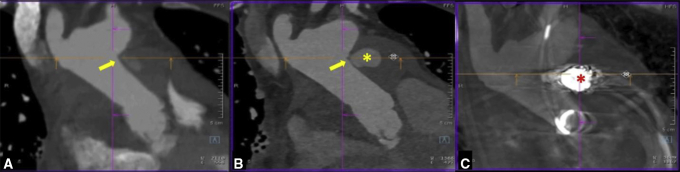
Embolization of the ascending aortic pseudoaneurysm. A, Initial cardiac computed tomography angiography on re-admission demonstrated a 4-cm ascending aortic pseudoaneurysm (*yellow arrow*). B, Repeat imaging after attempted endovascular embolization demonstrated interval increase in size with more contrast extravasation (*yellow asterisk*) in the periaortic region. C, Following percutaneous embolization under image fusion guidance, cone-beam computed tomography with contrast injection showed no extravasation or residual contrast filling of the pseudoaneurysm, and a coil mass (*red asterisk*) overlapping the region of original contrast extravasation.

Intraoperative cone-beam computed tomography (CBCT) (DynaCT; Siemens Healthineers) was performed after 200-degree C-arm rotation around the patient. Multiplanar reconstructions of CBCT were then fused with preoperative CCTA to identify the exact location of the AAP within the thorax. Using a dedicated needle guidance software (*syngo* Needle Guidance; Siemens Healthineers), a virtual needle path was planned through the intercostal space between the second and third ribs, away from the internal thoracic artery, lung parenchyma, and other vital structures, directly into the AAP (Figure 3). A dedicated needle guide (SeeStar; AprioMed AB) was positioned on the patient's chest using laser cross-hair guidance for percutaneous access based on the virtual needle path.

**Figure 3:**
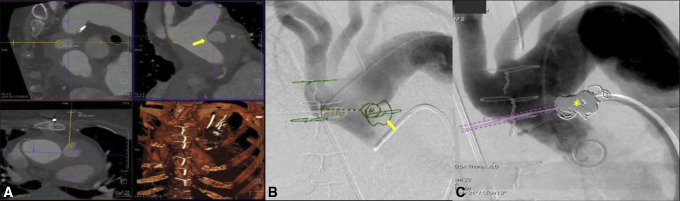
Planned virtual needle path. A, Preprocedural cardiac computed tomography angiography (CCTA) was used to map a needle trajectory into the ascending aortic pseudoaneurysm (*arrow*), avoiding vital structures. B, The CCTA map demonstrating the location of the pseudoaneurysm and other anatomical landmarks is fused with live fluoroscopy to allow needle insertion under supervision. C, Completion angiography demonstrates exclusion of the pseudoaneurysm by coil embolization (*asterisk*) deployed through a percutaneous needle (highlighted by pink tracks).

Femoral arterial access was obtained, and aortic arch angiogram was performed to confirm the location of the AAP, as mapped by CBCT-CCTA fusion imaging, and the accuracy of the virtual needle path. An 18-gauge needle (Chiba Biopsy Needle, Cook Medical) was then advanced into the AAP under live fluoroscopy at multiple C-arm angulations overlaid with the virtual needle path from CBCT using image fusion guidance, with immediate return of blood. Two-dimensional angiography was also performed to confirm proper needle position. Eight 0.035-inch coils (Interlock-35 Fibered IDC Occlusion System; Boston Scientific) were then deployed to embolize the AAP under live fluoroscopy and image fusion guidance. Subsequent aortic arch angiogram using digital subtraction angiography and CBCT with contrast injection showed no extravasation or residual contrast filling of the AAP, and a coil mass overlapping the region of original contrast extravasation (Figure 2, *C*).

Follow-up CCTA at the 1- and 2-year mark showed complete resolution of the APP in this patient.

## Discussion

The incidence of AAP ranges from less than 1% in the general population to roughly 13% in patients undergoing surveillance after cardiac or aortic surgery.^3^ AAPs develop postoperatively due to suture dehiscence or inherent weakness of the aortic wall layers.^1^^,^^2^ Open surgical repair is the standard of care but can be associated with high morbidity and mortality early after the index surgery, particularly in patients with multiple comorbidities.^2^ Less-invasive treatment options have been explored and used successfully, including endovascular approaches such as covered stent grafts, coil embolization, septal occluder devices, and vascular plugs.^3^ We present a novel approach to percutaneous coil embolization of an APP in a patient who was high risk for surgical repair and without suitable anatomy for an endovascular approach. The authors attest they are in compliance with human studies committee's regulations of the authors' institutions and Food and Drug Administration guidelines, including patient consent requirements.

## Conclusions

Contemporary hybrid operating rooms equipped with a C-arm and a robotic-assisted angiographic imaging system allow for intraoperative CBCT and image fusion with preoperative CCTA. During complex cardiovascular interventions, such image-fusion techniques allow integrated 3-dimensional imaging and visualization of target structures not typically visible on conventional fluoroscopy, thus improving procedural safety while reducing procedure time and radiation exposure.^5^ Direct, percutaneous, transthoracic coil embolization is an alternative treatment strategy for those patients who are not candidates for surgical or endovascular repair.

## Supplementary Data

Video 1 Procedural video of the planning and percutaneous, transthoracic coil-embolization of an ascending aortic pseudoaneurysm. Video available at: https://www.jtcvs.org/article/S2666-2507(22)00199-7/fulltext.
