# Supplementary figures and images for: Image fusion–guided percutaneous transthoracic embolization of ascending aortic pseudoaneurysm
Source: JTCVS Tech. 2022 Apr 12;13:1–3. doi: 10.1016/j.xjtc.2022.03.006 (PMC9196943; doi:10.1016/j.xjtc.2022.03.006)

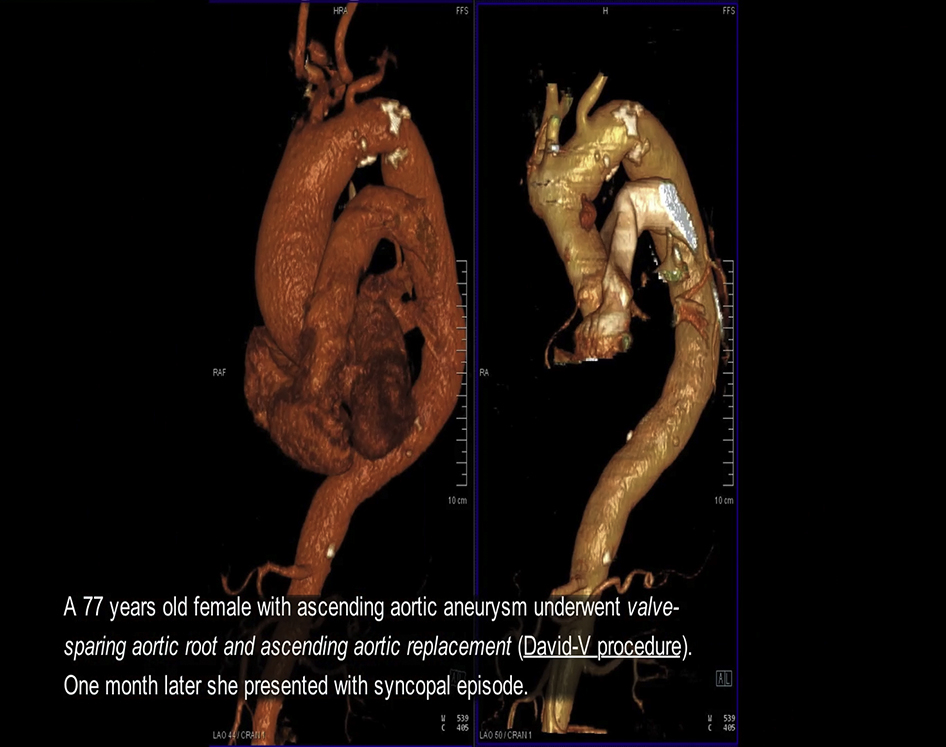

Supplement: Video 1 — Procedural video of the planning and percutaneous, transthoracic coil-embolization of an ascending aortic pseudoaneurysm. Video available at: https://www.jtcvs.org/article/S2666-2507(22)00199-7/fulltext. [file fx2.jpg]
